# Security Enhancement Mechanism Based on Contextual Authentication and Role Analysis for 2G-RFID Systems

**DOI:** 10.3390/s110706743

**Published:** 2011-06-28

**Authors:** Wan Tang, Min Chen, Jin Ni, Ximin Yang

**Affiliations:** 1 Computer Intelligence Lab, College of Computer Science, South-Central University for Nationalities, Wuhan 430074, China; E-Mail: tangwan@scuec.edu.cn; 2 School of Computer Science and Engineering, Seoul National University, Seoul 151-742, Korea; 3 School of Computer Science and Technology, Huazhong University of Science and Technology, Wuhan 430074, China; 4 School of Physics and Electronics, Henan University, Kaifeng 475004, China; E-Mail: lilynee822@gmail.com

**Keywords:** radio frequency identification (RFID), context-aware computing, role-based access control (RBAC), mobile code

## Abstract

The traditional Radio Frequency Identification (RFID) system, in which the information maintained in tags is passive and static, has no intelligent decision-making ability to suit application and environment dynamics. The Second-Generation RFID (2G-RFID) system, referred as 2G-RFID-sys, is an evolution of the traditional RFID system to ensure better quality of service in future networks. Due to the openness of the active mobile codes in the 2G-RFID system, the realization of conveying intelligence brings a critical issue: how can we make sure the backend system will interpret and execute mobile codes in the right way without misuse so as to avoid malicious attacks? To address this issue, this paper expands the concept of Role-Based Access Control (RBAC) by introducing context-aware computing, and then designs a secure middleware for backend systems, named Two-Level Security Enhancement Mechanism or 2L-SEM, in order to ensure the usability and validity of the mobile code through contextual authentication and role analysis. According to the given contextual restrictions, 2L-SEM can filtrate the illegal and invalid mobile codes contained in tags. Finally, a reference architecture and its typical application are given to illustrate the implementation of 2L-SEM in a 2G-RFID system, along with the simulation results to evaluate how the proposed mechanism can guarantee secure execution of mobile codes for the system.

## 1. Introduction

The Internet of Things (IoT) is intended to be rich in context awareness, and realized by various object sensing and information gathering technologies, such as Radio Frequency Identification (RFID), Wireless Sensor Networks (WSNs), etc. As the main technology for conveying the terminal information of object identification, the RFID system is one of main components of IoT. In [1], the traditional RFID system is referred as the First-Generation RFID System (1G-RFID-Sys). However, the information stored in the RFID tags (The Generation 2 (Gen-2) tag can be used [2], which is far superior to the Gen-1 version offering reduced inter-reader interference, faster read/write speeds, higher levels of data encryption, longer battery life and more data storage.) is intrinsically passive, static and non-intelligent. Consequently, the 1G-RFID-Sys neither suits the dynamics of the IoT environment nor has the intelligent decision-making ability to enable smart services with quick response. Therefore, Chen *et al*. proposed an evolution of 1G-RFID-Sys to a second-generation RFID system [1,3], referred as 2G-RFID-Sys (This is not to be confused with the Gen-2 RFID tag). The 2G-RFID-Sys tag contains not only static information, such as object identification and description, but also “mobile codes”. Mobile code is a type of encoded procedural directive and capable of intelligently “notifying” the system what operations or services should be provided in special occasions. The use of mobile code enables the 2G-RFID-Sys to be more extendible, reliable, and intelligent than the 1G-RFID-Sys.

Though the system extendibility and practicability can be effectively improved in a mobile and ubiquitous environment, the implementation of the intelligence conveyed in 2G-RFID-Sys brings a critical security issue due to the openness of mobile code. For example, how to prevent mobile code from being misused or abused in order to avoid malicious attacks which cause disruption of the Backend System (BS).

To address this issue, we extend our previous work [4] and introduce context-aware computing to expand Role-Based Access Control (RBAC) [5], while categorizing tags by role, *i.e.*, one tag belongs to one user and different users can be categorized by role. Assume each user is rational and follows the behaviorial pattern of location-aware constraints within a certain time interval. Mobile code is the hidden representation of user’s requirements. In order to categorize the tags in which the mobile code is stored, we actually divide users into different categories of roles. In this paper, the role is simply determined by several kinds of context-aware information, such as time, location, and historic service requested by mobile code. Based on the roles categorized according to contextual restrictions, we propose a Two-Level Security Enhancement Mechanism (2L-SEM) to prevent the misuse and abuse of mobile code, and eliminate illegal and invalid mobile codes in 2G-RFID-Sys. 2L-SEM operates in two phases as follows:

- *Contextual authentication*: A user needs to write some codes into the RFID tag informing the system what kinds of services he/she wants the system to provide. However, some user with ill intentions will inject a virus, rather than write service codes. To solve this problem, on the reception of a mobile code from the RFID reader, the information is verified in order to ensure it be satisfied pre-existing rules based on contextual constraints in BS authentication where the code is interpreted, otherwise, the code cannot be executed.
- *Role analysis*: Though some intrusive users can replicate mobile codes to pass through the authentication phase, the contextual information of the malicious user does not correspond to the role of the legal user, thus, the system can identify such malicious user and protect the legal user. Even if a code itself is acceptable to the system, once the “immediate role” has conflict with the “profile-based role”, the BS will prevent executing the code, *i.e.*, reject the services requested by the mobile code, and any following communications cannot be activated. Note that the roles in our mechanism are not the same as the environment ones defined in the system as in [5]. By comparison, they can be changed dynamically with the context-aware environment in which objects are involved. Accordingly, the 2G-RFID-Sys can adjust a subject’s role dynamically, and thus restrain the role’s action capacity to reduce the workload of role management.

The rest of this paper is organized as follows. We introduce related works in Section 2. Section 3 presents the security issues for 2G-RFID-Sys. In Section 4, taking into account the location and time usability, the restrictions for the usability and validity of mobile code are discussed in detail, in addition, the 2L-SEM is proposed. Section 5 describes and evaluates a distributed security solution for the 2L-SEM-based system through a special application case. Finally, Section 6 concludes this paper with some suggestions for future work.

## 2. Related Works

Typically, an RFID tag broadcasts the contained information to an RFID reader without certifying the reader’s identity, which is prone to information leakage and other security issues [6]. Recently, research has been mainly focused on the protection of data privacy in RFID tags. It shows that the well-used data security policies can guarantee information security for low-cost passive tags. In addition, RFID reader’s access to tag can be controlled through some schemes, such as mutual authentication [7], light-weighted cryptographic algorithms [8], collision-resolution protocols [9], blocking technology [10], *etc*., which can ensure the integrity and credibility of tag information with minimum information leakage and legality of tag owners. Zhou *et al.* proposed the Smart RFID Keeper (SRK), which controls reader’s unauthorized access to tags based on blocking technology [11]. Canard *et al.* presented the first security model for RFID authentication/identification privacy-preserving systems [12]. Rieback *et al.* proposed an effective scheme to enhance the security and privacy about passive RFID tags [13]. A lightweight privacy-preserving authentication protocol for RFID systems was proposed by E. Blass *et al.* [14]. The protocol uses a simple and round-based setup to let tags send the results and random nonces using small fan-in functions to the reader.

In the ubiquitous environment, applications are context-aware and user-centric [15,16]. Context-aware computing is an ability of these applications to detect and react to the various environments [17]. There are four types of context, *i.e.*, location, identity, time and activity. Burstein *et al.* applied mobile agents to the highly dynamic and variable context of the healthcare emergency decision-support domain [18,19], however, location awareness was not implemented. In [20], the RFID-based Campus Context-Aware Notification System (R-CCANS) was proposed to deliver notification to intended recipients in timely manner. R-CCANS uses contextual attributes of users in inferring the right notification to display, and then acts autonomously while the context is detected and processed. However, these actions are achieved passively from BS according to the tag context, such as the tag ID and location ID.

RBAC is an approach to restricting system access to authorized users, and sometimes referred as role-based security [21]. RBAC allows policies to be specified in terms of subject roles, each of which can be viewed as a set of subjects with the same permissions, rather than a set of individual subject identities [22]. In [5], K. Lee *et al.* proposed a context-aware security model for the dynamic environment based on multiple authentications, Multi-Attribute Utility Theory (MAUT), and extended Generalized RBAC (GRBAC). In the security model, the output of GRBAC is calculated according to the contextual information of tag, and then is adopted to MAUT as a utility value to calculate the security level of the user/tag. Based on the security level, the corresponding actions will be authorized by the BS.

With encoded rules that are stored as mobile codes in RFID tag, the capability of 2G-RFID-Sys is enhanced in order to act upon context-aware changes, and adapt services according to the dynamics of networks and end-user requirements. However, most of research pays more attentions to on-tag and off-tag security protection strategies between tag and reader, which is just suitable for static tag information protection, but inefficient for eliminating potential security hazards in 2G-RFID-Sys. In some other research, tag provides both static and active information of the user to the system based on RBAC, and achieve service from provider passively, that cannot take advantage of the mobile codes’ property in 2G-RFID-Sys. An optional module of the 2G-RFID reader, called identification filter (ID-filter), is used to check the ID information of tags. It provides security by maintaining a list of IDs that represents the validity of tags [1]. However, the security service provided by the simple ID-filter is limited, and a security enhancement mechanism should be designed to improve the security capability of BS.

The main functional components of 2G-RFID-Sys are RFID reader, BS and service response system. The RFID reader consists of a passive information manager, a code information manager, and an ID-Filter. The passive information manager receives identification and description information from the RFID tags, and the ID-filter checks the information through a list of IDs. The BS consists of an Electronic Product Code (EPC) network and a middleware layer. The information of tag approved through the ID-filter will be forwarded to the EPC network to create a record of the tag [1].

## 3. Security Requirement in 2G-RFID System

Although the architecture and archetypal application of 2G-RFID-Sys have been presented in previous work [1], the security issues are still in cloud. In this paper, we consider the security requirements in terms of two aspects, namely, static information security (denoted by static-info-security) and mobile code security (denoted by code-security). Currently, most research is focused on static-info-security, such as data and privacy protection, which are commonly used in 1G-RFID-Sys. This paper briefly addresses the security issue of 2G-RFID-Sys for the view of mobile code, such that legal codes are only executed by the authorized BS and evaluated in terms of validity.

The validity of a mobile code is closely related to the context when the mobile code is received, interpreted and executed. For instance, it will be affected by the tag writer, and the activity time and location of the mobile code. In a distributed and mobile network, a user writes some codes into the RFID tag informing the system of the kind of service he/she wants the system to provide. However, some user with bad intentions may inject a virus rather than write service codes, which will be addressed in the following sections.

## 4. Two-Level Security Enhancement Mechanism (2L-SEM)

In this section, we first provide some presentations of the contextual restrictions, and then propose 2L-SEM based on expanded RBAC.

### 4.1. Contextual Restriction

By means of the restricted usability of mobile code, it is guaranteed that the BS interprets and executes the right mobile codes, while pernicious codes are prevented. To identify the usability and validity of mobile code, this paper defines two types of contextual restrictions, *i.e.*, location restriction and time restriction, as shown below.

#### (1) Location Restriction

Location restriction is the constraint for the location validity of mobile code. In this paper, location validity indicates whether a mobile code can be activated, and is related to the present position of the RFID tag that maintains the mobile code and belongs to a legal user. That is to say, if the location is other than the user’s usual location in a non-authorized area, the mobile code cannot be activated by any BS, even if it is authenticated and usable.

Let ℂ*_L_* be the location restriction set, if (1):

(1) $$\forall m\in 𝕄[\exists l\in {\mathbb{C}}_{L}\Rightarrow (m,l)\in 𝕄\times {\mathbb{C}}_{L}]$$

is satisfied, mobile code *m* is available at location *l*. Thus, the function of location restriction for the mobile codes in set 𝕄 can be defined as (2):

(2) $$\text{Locations}(m\in 𝕄)=\{c|c\in {\mathbb{C}}_{L}\wedge (m,c)\in 𝕄\times {\mathbb{C}}_{L}\}$$

It should be noted that the location restriction is independent to the specific location definitions.

#### (2) Time Restriction

Time restriction is the constraint that is placed on the number of activities recorded and the activated time of a mobile code. The mobile code cannot be activated if its captured time does not satisfy the time restriction, or it has been activated more times than that allowed by the time restriction. There are two types of time restrictions, namely, continuous time restriction and periodic time restriction. If the activated time is constrained within a continuous time interval, it treats the continuous time interval as a continuous time restriction, otherwise, if the activated time is periodic in a pre-given time interval, the period is a time restriction for the mobile code.

**Figure 1.**
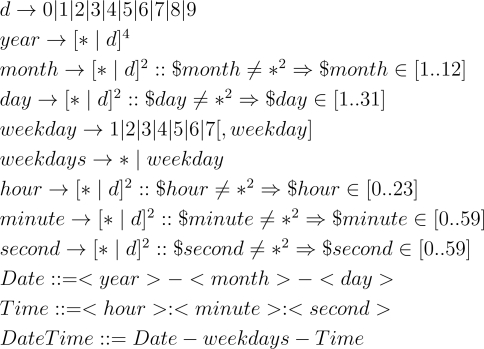
Time variable construction grammar.

Prior to giving the definition of time restriction, the time variable is defined based on a time pattern, which consists of some components (year, month, day, hour, second, *etc.*) and combination form (*i.e.*, DateTime) of time variable. The time pattern is represented via the time variable construction grammar defined in Figure 1. In the grammar, if a sub-variable (*i.e.*, a field of time variable) is a “*” string, it means that the sub-variable can be an arbitrary legal value. To illustrate the grammar clearly, some time variables and their values are given in Table 1. Consequently, we define a time range variable as an ordered pair < *t*_1_, *t*_2_ > composed of two time variables *t*_1_ and *t*_2_, and the time judging function InTimeRange is defined as (3). If the time variable *t^υ^* is within the time range < *t*_1_, *t*_2_ >, then the value of function InTimeRange is True, otherwise, it is False.

(3) $$\text{InTimeRange}(t^{\upsilon },<t_{1},t_{2}>)=\{\begin{array}{ll} \text{Ture} & t^{\upsilon }\geq t_{1}\wedge t^{\upsilon }\leq t_{2}\\ \text{False} & \mathrm{others} \end{array}$$

Table 2 gives some instances to illustrate the relationship between the function InTimeRange and variables. For example, a valid time interval, constructed by the time range variable given in the 3th row, is from Monday to Tuesday or from Friday to next Tuesday in December 2009, and the value of the time variable (2009-12-30-3-12:35:45) is not within the time interval, thus, the value of InTimeRange is False. However, the value of the time variable (2010-02-01-1-12:35:45) in the last row is not in the valid time range from December to next January in each year, therefore, the value of the time judging function is False.

We denote by *Count* the maximum times that a mobile code can be activated within a duration time, which is from time *t*_1_ to *t*_2_. Therefore, according to the previous definition, where the time restriction consists of a maximum number of activities recorded and an activated time for the mobile code, the time restriction pattern for the mobile code can be denoted as < *t*_1_, *t*_2_, *Count* >. We also denote the time restriction set by ℂ*_T_*. If (4):

(4) $$\forall m\in 𝕄(\exists c\in {\mathbb{C}}_{T}\Rightarrow (m,c)\in 𝕄\times {\mathbb{C}}_{T}\text{)}$$

is satisfied, then the mobile code *m* will be activated for at the most *Count* times within the given time range < *t*_1_, *t*_2_ > under the time restriction *c*. Therefore, the time restriction function of the mobile code *m* can be defined as (5):

(5) $$\text{ActTime}(m\in 𝕄)=\{c|c\in {\mathbb{C}}_{T}\wedge (m,c)\in 𝕄\times {\mathbb{C}}_{T}\}$$

### 4.2. Security Enhancement Mechanism

In this section, this paper designs a BS secure middleware, or 2L-SEM, to ensure the usability and validity of mobile code through two phases: *contextual authentication* and *role analysis*. Since a tag belongs to a user and the mobile code is the representation of the hidden representation of user’s requirements, the paper categorizes different tags by role. Because the associated roles with access privileges only changes infrequently within organizations relative to users, they can provide a stable and reliable control policy for the 2G-RFID-Sys based on the expanded RBAC.

#### (1) Contextual Authentication

This phase authenticates the usability of the mobile code delivered from the 2G-RFID reader. The usability is determined by several types of information of the mobile code, such as who wrote it, when and where it was written, and its valid period. Upon the reception of the mobile code, the information is verified to ensure that the pre-existing conditions/rules are satisfied based on the contextual constraints in BS. After passing the authentication by BS, the code is acceptable, otherwise, it is refused to be executed.

It is assumed that each role corresponds to a logical RFID tag group. The tags belonging to the same tag group play the same role and have similar mobile code sets. Each tag can play one or more roles, and each role can be played by one or more tags. Tag and mobile code are associated through role, and the relationships between these objects (*i.e.*, tag, role, and mobile code) are many-to-many mapping. Let 𝕋, ℝ, and 𝕄 be a non-empty finite tag set, a non-empty finite role set, and a non-empty finite mobile code set, respectively. Let *t* be a tag, *r* be a role, and *m* be a mobile code. If (6) is satisfied, the mobile code *m* maintained in tag *t* belongs to role *r*, and then it is valid to the BS.

(6) ∀(t∈𝕋∧r∈ℝ∧m∈𝕄)∃[(t,r)∈𝕋×ℝ∧(r,m)∈ℝ×𝕄]

Thus, for corresponding mobile code usability, this paper formally defines a restriction model as a 5-tuple (𝕋, ℝ, 𝕄, ℂ, *f*), where ℂ is the set of authorization and restriction rules associated with the special application system, *f* is the mapping from the tag set 𝕋 to the mobile code set 𝕄 and constrained by ℂ. The location restriction set ℂ*_L_* and time restriction set ℂ*_T_*, defined in Section 4.1, are both the subsets of ℂ. The constrained mapping *f* in the 5-tuple is described as below:

(7) f(t∈𝕋)={m|∃[(r,m,c)∈ℝ×𝕄×ℂ][(t,r)∈𝕋×ℝ∧(r,m)∈ℝ×𝕄]}

#### (2) Role Analysis

The role analysis checks whether the mobile code is a legal and valid member of a role or not. Even though a mobile code has gone through the authentication phrase, the tag in which the code is stored will be invalid or inactive for the system. For instance, two users with the same tag ID are waiting for service requested by the mobile code at the same time at different locations. If one of them is an invalid member of the role, the BS will not execute the code, and any following communication cannot be established. Fortunately, in the 2L-SEM, the validity of mobile code is ensured via the role analysis for tag. If a tag goes through the role analysis, it will be activated and obtain a permission that is the authorization of mobile code execution for a role. That is to say, the mobile code is a legal and valid member of the role, and will be accepted by the system and executed in the BS.

Before describing the policy of role analysis in 2L-SEM, we categorize the tag state 𝕋*_state_*, which is used to indicate the tag’s active state in the system, into three types, *i.e.*, attendee, actor, and absentee. Let 𝕋 denote a non-empty finite tag set, *SoT* denote the state of the tag, and then

$$\mathrm{SoT}(t\in 𝕋)\in {𝕋}_{\mathrm{state}}=\{\mathrm{attendee},\mathrm{actor},\mathrm{absentee}\}$$

Expanding the concept in RABC, we then classify the membership of roles into three types, *i.e.*, potential member or pMember, admissive member or aMember, and valid member or vMember. Let *t* be a tag, *r* be a role, and *c* be a contextual restriction rule maintained in BS. Assume the sets ℝ, 𝕄, 𝕋, and ℂ are the same as that defined in the proposed 5-tuple restriction model. The three types of role memberships are defined as follows.

(8) pMember(r∈ℝ)={t|t∈𝕋∧(r,t)∈ℝ×𝕋}

(9) $$\text{aMember}(r\in \mathbb{R})=\{t|\wedge \left\{\begin{array}{c} t\in \text{pMember}(r)\\ \text{SoT}(t)=\mathrm{attendee} \end{array}\right\}$$

(10) $$\text{vMember}(r\in \mathbb{R})=\{t|\wedge \left\{\begin{array}{l} u\in \text{aMember}(r)\\ \text{SoT}(t)=\mathrm{actor}\\ \forall (r,c)\in \mathbb{R}\times \mathbb{C}\Rightarrow t≺(r,c) \end{array}\right\}$$

where *t* ≺ (*r*, *c*) means that tag *t* satisfies restriction *c* of role *r*.

As a potential member of role *r*, tag *t* is also an admissive member of role *r*, only when it is an *attendee* of system. If its state is *actor* and information matches the restrictions of role *r*, tag *t* is also a valid member of role *r*, and can go through role analysis, as described by (11).

(11) Member(r∈ℝ)=aMember(r)∪vMember(r)⊆pMember(r)⊆𝕋

In the 2L-SEM, a tag cannot be an admissive member and a valid one at the same time.

(12) ∀t∈𝕋,r∈ℝ⇒¬(t∈aMember(r)∧t∈vMember(r))

## 5. Reference Architecture and Its Application

A 2L-SEM-based reference architecture is designed for 2G-RFID-Sys in this section. In addition, a highway management system is provided as an application case in order to illustrate how the proposed 2L-SEM can be applied and how it guarantees the system security and performance.

### 5.1. Reference Architecture

**Figure 2.**
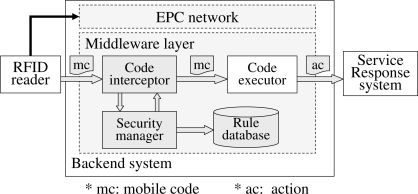
Reference architecture for 2L-SEM-based system.

In this subsection, we design a 2L-SEM-based reference architecture for the 2G-RFID-Sys. In the reference architecture shown in Figure 2, the code interceptor is responsible for constructing audit requests relying on the context of a mobile code delivered from an RFID reader, and applies these audit requests to the security manager. Then, the security manager will operate our security enhance middleware, *i.e.*, 2L-SEM, to make a judgment regarding the request by searching the authorization and restriction rules stored in the rule database. If an authority exists and the request is in accordance with the rules, then the security manager returns a permission message, otherwise, it denies the request of the mobile code execution. While the code interceptor receives a permission massage sent by the security manager, the corresponding mobile code will be forwarded to the code executor which is corresponding to the middleware layer in the original 2G-RFID BS.

Each application of 2G-RFID-Sys is composed of several management areas. For each area, we allocate a security manager or a security manager group equipped with a rule subset, as an effective way to reduce the time consumption for the identification of a valid request. However, since the security managers should cooperate with each other to make a judgment, the system performance will be degraded. Furthermore, the 2L-SEM-based system can add a component to notify security managers to overload the rule subset when changes occur.

### 5.2. System Instance

In the highway management application, a 2G-RFID-Sys tag is carried by a vehicle, which is a private car but not an ambulance, and stores a mobile code MC_001: “emergency: on”. While the car is passing through a speed detection point equipped with an RFID reader on the highway, the availability of the mobile code decides whether it can be interpreted and executed by the BS. And then, the corresponding service or action will be provided.

Next, some examples are given in Table 3 to illustrate the mapping relationship between subjects and time restrictions. Each subject includes three items: tag, role and mobile code. Different tags (e.g., Tag_001 and Tag_002) with different rule sets are playing the same role Private Car, and the same mobile code (e.g., MC_001) is carried by different roles (e.g., Private Car and Ambulance). The time restriction consists of the time variable *StartTime,* the time variable *EndTime,* the maximum number of activities recorded, and the *DurationTime*. The mobile code playing as a role can be activated within a time range < *StartTime, EndTime* >. However, if the value of *StartTime* is Null, the activated time range is < *t_w_*, *EndTime* >, where *t_w_* is the time when the code was written in the tag. Furthermore, if the value of *EndTime* is also Null, the mobile code can be activated within the time range < *t_w_*, *t_w_* + *EndTime* >. Finally, if the value of *DurationTime* is 0, the mobile code can be activated at anytime.

As shown in Table 3, if the tag is carried by a private car, MC_001 can only be activated during six hours. However, it is available at anytime if it is stored in an ambulance tag. As mentioned above, the mobile code MC_001 can be written not only in an ambulance’s tag, but also in a private car’s tag. The driver of a private car is subject to an emergency situation while transporting a pregnant woman to the hospital, and then MC_001 is written in the tag carried by the car at 5:15 am of 30 November 2010. After one day, when the car passes a speed detection point. The mobile code is delivered to the BS in the highway speed detection system and goes through the authentication phase. However, MC_001 cannot be activated since it is not maintained by an ambulance’s tag, but a private car’s tag whose duration time cannot exceed six hours.

### 5.3. Processing Sequence

**Figure 3.**
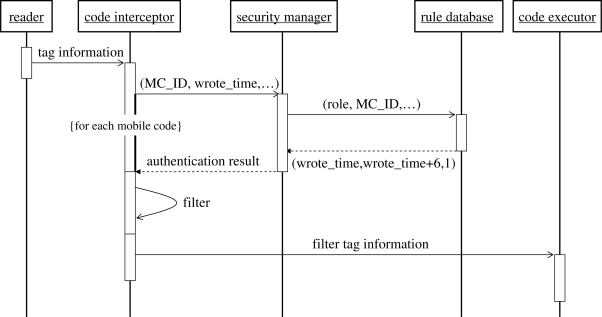
Processing sequence of 2L-SEM-based system.

Relative to the system instance, while the overspeed vehicle is carrying patient in a critical and emergency situation, the code interceptor in the reference architecture will receive a permission for executing the mobile code, and then deliver the entire information contained in the tag to BS. Otherwise, the code interceptor will shield the mobile code, and only deliver the normal tag information (e.g., RFID type, identification, and object description) to BS. Consequently, the BS could dispose of the overspeed vehicle correctly. The processing sequence is described in Figure 3.

Assume that there is only one time restriction, e.g., a six-hour active time for the mobile code MC_001. Having received the mobile code, the code interceptor encapsulates a request with the time-related context information in the form of *(BS_ID, MC_ID, ExecutingTime)*, and sends the request to the security manager. The rule

(Role_any,MC_ID,wrote_time,wrote_time+6,−1)

as searched by the security manager, indicates that the mobile code with identification *MC_ID* is effective for any role during six hours after being written into an RFID tag. If (*ExecutingTime* ≤ *wrote_time* + 6) is satisfied, the security manager will send back a permission message to the code interceptor, otherwise, a denying message will be released. According to the message returned by the security manager, the code interceptor may filter the rejected mobile code from the tag, and then pass forward the tag information to the code executor for subsequent work. Moreover, illegal mobile codes can also be eliminated by the interceptor, which is able to effectively protect the BS from the damage caused by malicious mobile codes.

### 5.4. Performance Evaluation

**Figure 4.**
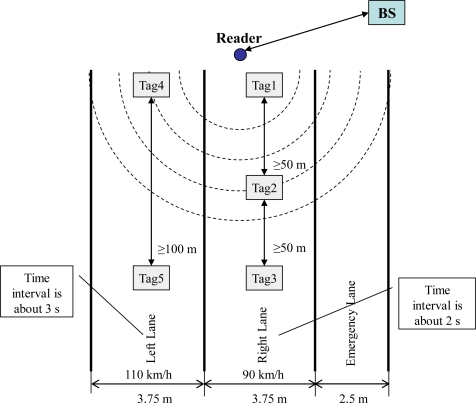
Highway simulation scenario.

In order to evaluate the proposed 2L-SEM, we established a simulation platform for the 2L-SEM-based highway speed detection system using SimJava 2.0 [23]. In the simulation scenario shown in Figure 4, there are three one-way lanes: Emergency Lane, Left Lane which is used for overtaking, and Right Lane. The widths of the Left Lane and Right Lane are both 3.75 m. Each tag is corresponding to one vehicle. The read/write range of the reader is indicated using broken arc lines. On the Right Lane, the speed limit is 90 km/h and the minimum safety distance between vehicles is 50 m. On the Left Lane, the speed limit is 110 km/h and the minimum safety distance is 100 m. Normally, the speeds of vehicles on the Right Lane and the Left Lane are 80–100 km/h and 100–110 km/h, respectively.

It is obvious that the time interval between two vehicles is about 2 s on the Right Lane if their distance is equal to the minimum safety distance. In the same way, that is about 3 s on the Left Lane. In the scenario, there are no more than three vehicles (e.g., Tag1, Tag2 and Tag3) on the Right Lane between two vehicles (e.g., Tag4 and Tag5) on the Left Lane at a given time. The time intervals between Tag1 and Tag4, between Tag1 and Tag2, between Tag2 and Tag3, and between Tag3 and Tag5 are 0, 2, 2, and 0 s, respectively. From another point of view, there is the most one vehicle (e.g., Tag4) which is running on the Left Lane between two vehicles (e.g., Tag1 and Tag2) on the Right Lane at the same time. Each time interval among these three vehicles ranges from 0 to 2 s.

According to the scenario described above, we assume that the time interval between tags arriving at the reader follows an exponential distribution with a mean *r*. The number of mobile codes in each tag *n_t_* is in the uniform distribution on the interval [0, 20]. We ran the platform five times with different tag time intervals with mean *r* (= 2, 4, 5, 8, and 20 s) to simulate the actual situation on the highway.

**Figure 5.**
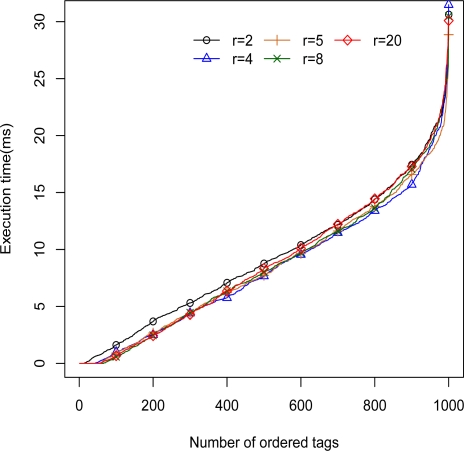
Distribution of the tags execution time.

**Figure 6.**
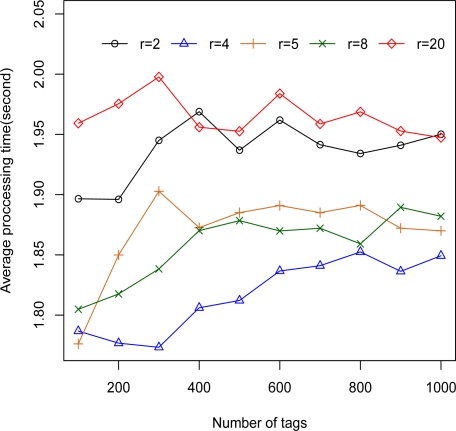
Average tag processing time.

**Figure 7.**
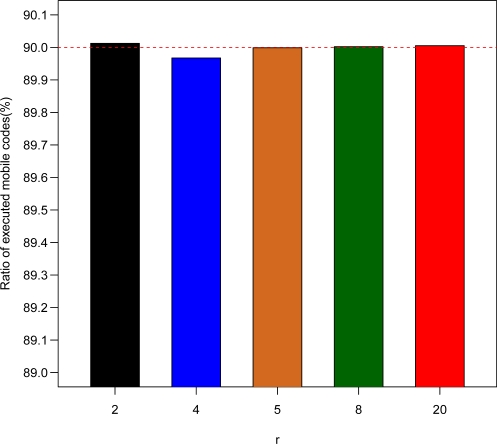
Ratio of executed mobile codes.

In order to test the performance and capability of the 2L-SEM-based 2G-RFID system, the circumstances of these simulations are worse than normal. Firstly, the hardware performance is very low. For a mobile code, the average service time of the rule database *t_d_* obeys the exponential distribution with mean 0.2 s, and the average execution time of code executor *t_e_* is 1 ms. We then assume the number of mobile codes provided by system *n_m_* is 100, which is much more than that needed in the real case. Finally, the ratio of authenticated mobile codes *p_a_* is 90%. The performance is evaluated in terms of tag execution time, average tag processing time, and rate of mobile codes execution, with the results shown in Figures 5–7.

First, the distribution of the tags execution time consumed in the code executor is depicted in Figure 5. Based on the reference architecture given in Section 5.1, the average execution time of tags should be ((20/2) * *p_a_* * *t_e_* =)9 ms, and the maximum execution time of tags should be (20 * *t_e_* =) 20 ms. In all the five cases with different tag time intervals, 95% and 51% tags are executed within 20 and 9 ms, respectively. The 2L-SEM-based application case can achieve the desired performance, that is to say, the tag execution time of the system can be achieved via setting system parameters.

Hence, the efficiency of BS authorization and mobile code filtering in the 2L-SEM-based system is surveyed. We have measured the average processing time as a function of the number of tags under different time intervals of tags with mean *r* (= 2, 4, 5, 8, 20) on the order of second, and the results are given in Figure 6. As the number of tags increases, the average tag processing time approaches 1.9 s, which is approximating the average service time of the rule database. It is obvious that the efficiency of the designed system is decided by the service time of the rule database.

Subsequently, we analyze the validity of the mobile code execution according to the results given in Figure 7. Nearly 90% of mobile codes carried by all tags are executed with the exception of case (*r* = 4). The ratio of authenticated mobile codes *p_a_* is 90%. It is indicated that all most authenticated mobile codes in the application case are executed.

To obtain insights into the performance of 2L-SEM under various average service time of rule database for one mobile code, we have also measured the performance in terms of processing time, length of waiting queue, and tag waiting time for processing. The results are given in Table 4. The arrival process of tags obeys the log-normal distribution. More specifically, with respect to the actual vehicle traffic on the highway, we vary the interval time with mean *r* = 0.01 and standard deviation *sd* = 0.2, so the most interval time between two adjacent tags is around 1 s, and avoids a number of short (<0.5 s) or long (>2 s) interval time. In our simulations, the number of mobile codes in one tag is between 0 to 20, and then the processing time of one mobile code is about 100 ms. Nevertheless, the processing time of a single tag can be 50 ms while the interval time is 1 s.

The performance of the 2L-SEM-based system decreases as the service time (or verification time) of rule database *t_rdbs_* increases. Especially, the performance will deteriorate if *t_rdbs_* exceeds 0.1 s. As shown in Table 4, when *t_rdbs_* is less than 40 ms, the maximum tag processing time is about 1 s, there is no more than one tag which is waiting for processing and its waiting time is less than 0.5 s. Thus, the performance is guaranteed. In the case that *t_rdbs_* is between 40 ms to 100 ms, the requirement of the application system is still met. According to special environment and requirement in reality, the 2L-SEM-based systems could keep the average service time of rule database *t_rdbs_* be less than 0.1 s via database self-optimization, e.g., partition, dynamic cache, and hierarchical architecture.

## 6. Conclusions

The scalability and flexibility of 2G-RFID-Sys will support more intelligent applications in the future. How to prevent mobile codes from being misused or abused and detect malicious mobile codes is a critical issue of the 2G-RFID-Sys. This paper addresses the code-security issue by the following three major contributions. First, location and time restrictions are defined to represent the set of authorization and restriction rules for special application system. Secondly, we propose a two-level security enhancement mechanism for 2G-RFID-Sys by expanding the restraint policy and concept of RBAC. Taking advantage of the context information, the mechanism can dynamically adjust a subject’s role, and restrain the action capacity of the role to reduce the workload of role management. Thirdly, the paper designs a reference architecture for the 2G-RFID-Sys based on the proposed mechanism. In addition, this paper presents an application case—a highway speed detection system—to illustrate and verify how the proposed mechanism can provide intelligent and secure information processes for 2G-RFID-Sys. In future, we will represent more contextual restrictions (e.g., action-profile restriction), implement context process, and demonstrate 2L-SEM in the testbed with the help of backend based on the Field Programmable Gate Array (FPGA) platform.

## Figures and Tables

**Table 1.** Examples for time variable.

**Time variable**	**Value**
2009-12-30-*-12:00:00	2009-12-30 12:00:00
2009-12-30-*-12:**:**	2009-12-30 12:00:00 -12:59:59
2009-12-**-1,3,5-**:**:**	Any time on every Monday, Wednesday and Friday in Dec. 2009
****-01-**-*-**:**:**	Any time in January in each year

**Table 2.** Relationship between variables and function InTimeRange.

**Time variable**	**Time range variable**	**Value**
2009-12-30-3-12:35:45	(2009-12-30-*-12:00:00, 2009-12-30-*-13:00:00)	True
2009-12-30-3-12:35:45	(2009-12-30-*-12:**:**, 2009-12-30-*-12:30:**)	False
2009-12-30-3-12:35:45	(2009-12-**-1,5-**:**:**, 2009-**-**-2-**:**:**)	False
2009-12-30-3-12:35:45	(****-1-**-*-**:**:**, ****-12-**-*-**:**:**)	True
2010-02-01-1-12:35:45	(****-12-**-*-**:**:**, ****-1-**-*-**:**:**)	False

**Table 3.** Mapping relationship between subject and time restriction.

**Tag**	**Role**	**MC***^a^*	**StartTime**	**EndTime**	**Mat***^b^*	**Dt***^c^*
Tag_001	Private Car	MC_001	Null	Null	0	6 hours
Tag_002	Private Car	MC_002	Null	2012-12-31-*-12:59:59	0	0
Tag_003	Ambulance	MC_001	Null	Null	0	0
Tag_004	Ambulance	MC_002	2010-9-1-*-00:00:00	2010-10-31-*-12:59:59	0	0
...	...	...	...	...	...	...

a Mobile code;

b Max number of activities recorded;

c DurationTime.

**Table 4.** Statistic results under different service time of rule database (s).

*t_rdbs_*	*et_min_*	*et*_avg_	*et_max_*	*ql_min_*	*ql_avg_*	*ql_max_*	*wt_min_*	*ql_avg_*	*wt_max_*
0.01	0.0003	0.1771	0.5241	0	0.004	1	0	0.0004	0.1148
0.02	0.0003	0.2866	0.8154	0	0.022	1	0	0.0017	0.2569
0.03	0.0003	0.3830	1.1155	0	0.061	1	0	0.0087	0.4539
0.04	0.0003	0.4919	1.4513	0	0.144	2	0	0.0320	0.7971
0.05	0.0003	0.6295	2.0787	0	0.236	3	0	0.0742	1.2397
0.06	0.0003	0.7705	2.4900	0	0.331	3	0	0.1338	2.0976
0.07	0.0003	0.9445	3.3102	0	0.466	3	0	0.2221	2.0030
0.08	0.0003	1.2873	4.8508	0	0.828	5	0	0.5057	4.0512
0.09	0.0003	1.4438	5.6199	0	0.866	5	0	0.5792	4.7174
0.10	0.0003	2.0405	8.7454	0	1.426	8	0	1.1033	7.4734
0.12	0.6512	24.2337	39.9250	0	20.827	36	0	23.0921	38.9538
0.15	0.8876	156.7548	319.2479	0	107.695	229	0	155.3244	318.6637

*t_rdbs_*: average service time of rule database; *et*: processing time of tags; *ql*: length of waiting queue; *wt*: waiting time for processing.
